# Supplementary material for: Are carbon emissions trading and green financial instruments synergistic? -Comprehensive quantitative research based on content analysis
Source: PLoS One. 2024 Mar 7;19(3):e0298601. doi: 10.1371/journal.pone.0298601 (PMC10919601; doi:10.1371/journal.pone.0298601)
Supplement: S1 Data — (ZIP) [file pone.0298601.s001.zip › Data/地级市Z_F工作报告.zip!/╣π╢1⁄2/╣π╓▌╩╨/└┤╫╘╣1⁄2╓┌║┼í╛┬φ┐╦╩2╛▌═°í┐_2019.docx]

各位代表：

我代表市人民政府，向大会报告政府工作，请予审议，并请政协各位委员和其他列席人员提出意见。

一、2018年工作回顾

去年，是全面贯彻党的十九大精神开局之年。3月7日，习近平总书记亲临全国人大广东代表团参加审议并发表重要讲话，对广东提出了“四个走在全国前列”的要求。10月下旬，总书记视察广东时进一步提出深化改革开放、推动高质量发展、提高发展平衡性和协调性、加强党的领导和党的建设的要求，强调广州要实现老城市新活力，着力在综合城市功能、城市文化综合实力、现代服务业、现代化国际化营商环境方面出新出彩。总书记的重要讲话为广州发展指明了前进方向，提供了根本遵循。

一年来，我们牢记习近平总书记的殷殷嘱托，坚持以习近平新时代中国特色社会主义思想为指导，按照党的十九大作出的战略部署，牢固树立“四个意识”，坚定“四个自信”，坚决做到“两个维护”，认真落实省委“1＋1＋9”工作部署和市委“1＋1＋4”工作举措，坚持稳中求进工作总基调，贯彻新发展理念，深化“大学习、深调研、真落实”工作，扎实抓好中央巡视和国务院大督查反馈问题整改，统筹推进稳增长、促改革、调结构、惠民生、防风险工作，积极稳就业、稳金融、稳外贸、稳外资、稳投资、稳预期，经济社会发展取得新进步。

去年，我市经济质量效益稳步提升，预计地区生产总值2.3万亿元，地方一般公共预算收入1632.3亿元、可比增长9.9％，高新技术企业数量突破1万家，一批重大产业项目进展顺利，10大价值创新园区加快发展。枢纽型网络城市建设加快推进，机场、港口、高铁、地铁等重大项目建设取得进展，承载力不断增强，广州在世界城市体系、全球科技创新实力、全球金融中心等排名明显提升。营商环境持续优化，行政审批和服务效率稳步提高，政务环境排名居全国前列。城市治理取得积极成效，拆违、治水力度空前，老旧小区微改造步伐加快，园林绿化景观更加靓丽，“四标四实”成果应用全面推开。人民生活水平不断提高，城乡居民人均可支配收入分别增长8％和10％，十件民生实事基本完成，房地产市场保持平稳，外围城区公共服务设施加快完善，市民群众的获得感、幸福感、安全感不断增强。主要做了以下工作：

（一）大力发展实体经济。面对复杂多变的国内外经济形势，我们保持战略定力，把工作着力点放在实体经济上，狠抓打基础、利长远工作，坚定不移推动经济高质量发展。

积极培育壮大新动能。出台系列产业扶持政策，加快发展新一代信息技术、人工智能、生物医药和新能源、新材料产业，重点培育6个新支柱产业集群，获批国家智能网联汽车与智慧交通应用示范区。新兴产业增加值增长7％，其中智能装备与机器人增长10.4％，新一代信息技术增长9.1％，轨道交通增长9％。先进制造业和高技术制造业增加值占规模以上工业比重分别为59.7％和12.8％，同比分别提升1.6个和0.1个百分点。做优做强现代服务业，建设国家绿色金融改革创新试验区，获批国家服务型制造示范城市。现代服务业增加值增长6.7％，占服务业比重66.5％，同比提升0.4个百分点，其中信息服务业增长25％以上，旅游业收入增长11％，新增上市公司10家、新三板挂牌企业21家。大力发展新零售等新兴消费，打造9个商旅文融合功能区，社会消费品零售总额9240亿元、增长7.5％，网上商店零售额增长15％。培育外贸新业态新模式，商品进出口总值9829亿元、增长1.2％，飞机融资租赁增长61％，邮轮进出港旅客增长19.3％。

推动传统产业转型升级。启动19个产业园区提质增效试点项目，92个项目获评国家级绿色制造示范项目，完成184户国有“僵尸企业”实质出清，规模以上工业企业利润增长8％。促进专业市场转型升级和有序疏解，安华汇、万菱广场等6家专业市场向国际化、展贸化、信息化转型。大力发展都市现代农业，农业增加值248亿元、增长3％，获批国家现代农业产业科技创新中心。加强质量、标准、品牌强市工作，新增13个国家级标准化示范试点项目。

促进有效投资增长。扎实推进重点项目建设，将398个重点项目纳入“攻城拔寨”行动，对184个影响大、关注度高的项目挂图作战，重点项目完成投资2812.8亿元，带动全社会固定资产投资增长8％，高技术制造业投资增长1.5倍，工业投资增长50％。瞄准重点企业精准招商，新设外商直接投资企业5376家、增长1.2倍，在穗投资的世界500强企业达301家，实际利用外资67亿美元、增长6.5％，其中制造业实际利用外资增长2.26倍。

加大营商环境改革力度。出台实施营商环境综合改革试点方案，企业开办时间压减至4个工作日以内，政府投资工程建设项目审批时间压减至90个工作日以内，社会投资项目压减至50个工作日以内，企业退税时间和清税申办时间大幅压减，口岸整体通关时间压缩三分之一，越秀区“企业开办最快1天”受国务院大督查通报表扬。深入推进放管服改革，下放各类事权123项，清理证明事项380余项，新登记市场主体和企业分别增长25.45％和35.25％。落实“民营经济20条”，民营经济增加值增长6.7％，民营经济制造业投资增长12.3％。率先推动部门整体预算绩效管理，形成全闭环管理机制。积极防范化解重大风险，银行机构不良贷款率1.05％，国有企业资产负债率59.2％，政府存量债务下降16％，风险总体可控。

（二）优化提升中心城市功能。对标国际一流城市，在全球资源配置体系中谋划、建设和发展，不断增强城市影响力、辐射力和显示度。

坚持规划引领发展。开展新一轮城市总体规划、土地利用总体规划编制试点，与审批制度改革相衔接的“多规合一”管理平台上线运行。在全国先行先试国土空间规划编制，制定交通发展战略规划和一批重大发展平台控制性规划。推进全国城市设计试点。精准配置土地资源，有效保障重点项目用地。推动国家历史建筑保护利用试点，开展历史文化名城保护规划实施评估和南粤古驿道重点线路建设。

提升科技创新能力。高水平推进再生医学与健康、南方海洋科学与工程省实验室建设，新增国家级创新平台15家、省级平台80家，广州高新区入选国家十大世界一流高科技园区。开展高新技术企业树标提质行动，新增高新技术企业超过2000家，科技创新企业超过20万家，国家科技型中小企业备案入库数居全国城市第一。设立50亿元科技成果产业化引导基金，科技信贷风险资金池拉动银行贷款超过100亿元，股权投资机构达到6192家。举办海交会、小蛮腰科技大会等系列活动。新发放“人才绿卡”1580张。世界技能大赛全国选拔赛11个项目获第1名。开展知识产权运用和保护综合改革试验，专利和发明专利授权量分别增长45％和20％，技术合同成交额增长1倍以上。

强化国际综合交通枢纽功能。推进18项国际航运枢纽工程建设，广州港深水航道拓宽工程一期投入使用，新增国际集装箱班轮航线12条，港口货物吞吐量6.12亿吨、增长3.7％，港口集装箱吞吐量2191万标箱、增长7.6％。推进11项国际航空枢纽工程建设，白云机场二号航站楼、商务航空服务基地等项目投入使用，空客、波音飞机客改货项目落户，新开及复航国际航线18条，机场旅客吞吐量6974.32万人次、增长5.9％，货邮吞吐量188.72万吨、增长5.9％。推进5个国铁、6个城际铁路、9个地铁和10个高速公路项目建设，白云（棠溪）站、镇龙站、金融城站等综合交通枢纽开工，6条（段）地铁新线开工建设，4条（段）地铁开通运营，实现所有行政区通地铁。推动100个市政路桥工程建设，北三环二期、凤凰山隧道、从化大桥建成通车，南沙大桥主线贯通，被授予国家公交都市建设示范城市。实施“四好农村路”建设三年行动计划，试行路长制。落实信息基础设施建设三年行动计划，光纤接入用户占比91.6％，累计建成充电桩2.6万个。

提高城市开放合作水平。抓住粤港澳大湾区建设重大历史机遇，研究制订规划纲要实施意见系列文件，推动各重点领域工作取得实效。建设南沙粤港澳全面合作示范区，与香港科技大学签订合作办学协议，香港赛马会从化马场建成使用，广深港高铁开通运行。深度参与“一带一路”建设，开行中欧班列65列，牵头成立举办海丝申遗城市联盟，新建10个旅游境外推广中心。加快南沙自贸试验区建设，新增改革创新成果105项，其中33项在全国、全省复制推广。开展庆祝改革开放40周年系列活动。举办世界航线发展大会、从都国际论坛、全球市长论坛等国际性会议61次，124届广交会参展企业2.56万家、出口成交额298.6亿美元，新增4个国际友好港、3家驻穗总领馆，友好关系城市增至73个。深化广佛同城化、广清一体化、穗莞战略合作，与中山、佛山港口资源合作取得进展。推动援疆援藏和梅州、清远、毕节、黔南、甘孜、巫山对口帮扶，加强齐齐哈尔对口合作，落实财政扶贫资金39.4亿元。

（三）加大城乡环境治理。牢固树立绿色发展理念，统筹城乡协调发展，持续推进建设干净整洁平安有序城市环境，以坚定的决心和有力举措加强生态环境保护，建设宜居宜业宜游的美丽广州。

打好污染防治攻坚战。铁腕整改中央环保督察反馈的1414件突出生态环境问题。关停广州发电厂、旺隆发电厂7台燃煤机组，推广应用纯电动公交车1.1万台，完成262家重点企业挥发性有机物综合治理。PM2.5平均浓度35微克/立方米，连续两年达到国家二级标准，未出现重污染天气。推动河长制湖长制见行动见成效，清理整治“散乱污”场所6万个，其中关停取缔3.6万个。全面完成52个集中式饮用水水源地环境整治，北部水厂建成通水。建成污水处理厂3座，新建污水管网3430公里，基本完成48个城中村截污纳管主体工程建设。8个国考、省考断面水质达到年度考核要求，35条黑臭河涌长制久清，112条黑臭河涌治理主体工程完工，入选全国黑臭水体治理示范城市。

改善城乡环境面貌。大力推进城市更新改造，完成5个老旧小区改造国家级试点，77个老旧小区微改造、7条旧村改造、20个旧厂改造项目完工。推动创建71条美丽乡村和30个特色小镇、1个名镇、15个名村。开展农村人居环境综合整治，60％以上村达到省定干净整洁村标准，30％以上村达到省定美丽宜居村标准。完成16个污染地块土壤修复。强化固体废弃物处理，新建13座建筑废弃物消纳场，创建300个生活垃圾精准分类样板居住小区，建成兴丰应急填埋场一期，4座资源热力电厂试运营。擦亮花城绿城品牌，举办中国森林旅游节，种植主题花树10万株，新增立体绿化10万平方米、生态景观林带80公里，建成绿道100公里、森林公园2个、湿地公园1个，创建森林小镇3个，完成碳汇造林4.5万亩。

加强城市精细化管理。开展广州大道、人民桥等路桥品质提升工程和桥下空间整治。优化调整部分街道行政区划，规范命名城乡道路3200条。加大违法建设治理力度，拆除违法建设7.3万宗、3600万平方米。新改建公厕1027座，推动机关事业单位开放厕所1659间。新增管道燃气居民覆盖用户37.79万户。建立食品药品风险管控机制。整治安全生产隐患57.5万项。完善应急管理体系，生产安全事故起数、死亡人数分别下降21.1％和16.5％。实施非广州市籍中小客车“开四停四”管理措施，完成12个交通微改造工程，整治交通拥堵黑点26处，多措并举缓解交通拥堵压力。

（四）着力保障和改善民生。坚持以人民为中心的发展思想，加大民生投入，推动基本公共服务均等化、优质化，让改革发展成果更多更公平惠及全市人民。

织密织牢民生保障网。提升就业服务质量，城镇新增就业33.7万人，城镇登记失业率1.9％。持续推进社保扩面，累计发放社保卡1275万张，企业退休人员月均养老金提高至3513元。推行医疗机构药品集中采购，全面实施按病种分值付费，推进异地就医直接结算。加大住房用地供应，完善租购并举的住房制度，扩大户籍家庭住房保障覆盖面，基本建成保障性安居工程3.2万套，新增租赁住房110.4万平方米，成立5家国有住房租赁企业。完成旧楼宇加装电梯869宗。实施养老大配餐服务提升工程，长者饭堂增加到1002个。完成45个肉菜市场改造。兜住民生底线，每人每月低保标准提高到950元、城乡特困人员供养标准分别提高到1721元和1964元、孤儿养育标准提高到2263元，医疗救助108.91万人次，城乡居民医保政府资助标准每人每年提高至490元，城乡居民养老保险基础养老金政府补助标准每人每月提高至211元。资助9.7万名失能、独居、高龄老年人使用“平安通”，分类帮扶保障1.8万名困境儿童。

提高公共服务水平。落实市人大2号议案决议实施方案，推进58个市重点民生基础设施项目建设。实施学前教育和中小学校三年提升计划，新增学前教育学位3.89万个、中小学学位13.79万个，完成37所校园功能微改造，新增10所市示范性高中、3个市属教育集团，成为国家级教育综合改革实验区。3所技工院校纳入省高水平技师学院创建学校。华南理工大学国际校区开工建设。开展来穗人员系列关爱活动，积分制指标增加至7000个，新增来穗人员随迁子女中小学起始年级学位2.87万个。深化医药卫生体制改革，加快推进分级诊疗，网格化布局紧密型医疗联合体，全面取消公立医院医用耗材加成，打造高品质家庭医生服务受国务院大督查通报表扬。传承发扬岭南优秀传统文化，建设一批新时代红色文化讲堂，加快广州文化馆、美术馆、华侨博物馆建设，南汉二陵博物馆展陈工程完工，岭南金融博物馆建成开馆，荣获中国版权金奖，举办文化产业交易会、国际纪录片节等国际性节展活动，172个公共图书馆（分馆）实现通借通还，行政村（社区）综合性文化服务中心全覆盖，举办文化惠民活动2.8万多场。发展体育事业，亚运会16个项目获金牌，省运会获金牌、奖牌、总分三项第一，广州马拉松获评国际田联金标赛事，成功举办世界羽毛球巡回赛总决赛等大型赛事，承办中国体育文化博览会和中国体育旅游博览会。

加强和创新社会治理。开展创新城乡社区治理试点，实现村居议事厅全覆盖。基本完成农村土地承包经营权确权登记颁证任务。出台社会工作服务条例，新登记社会组织637个。深化“羊城慈善为民”行动，荣获第十届中华慈善奖，城市公益慈善指数排名全国第二。实现婚姻登记“全城通办”。完善社会治安立体化信息化防控体系，案件类警情、刑事立案分别下降14.2％和13.2％。深入开展扫黑除恶专项斗争，打掉涉黑团伙15个、恶势力犯罪集团36个，群众安全感满意度分别达98.4％和97.8％。开展出租屋“八查”行动，整治出租屋安全隐患57.3万处。开展系列宪法宣传教育活动，司法工作不断加强。

（五）全面提高政府效能。坚持为民情怀，加快政府职能转变，着力提升政务服务水平，增强政府执行力和公信力。

加强法治政府建设。依法依规向人大及其常委会报告工作，向政协通报情况。认真执行市人大水环境治理和保护议案决议、民生基础设施规划与建设议案决议，办理市人大代表重点建议4件、市政协重点提案6件、市政协建议案5件，办理各级人大代表建议415件、政协提案501件。认真听取民主党派、工商联、无党派人士及工会、共青团、妇联等人民团体意见。提请市人大常委会审议法规草案20件、制定修改政府规章22件，废止规章、规范性文件161件。坚持依法统计，确保数据真实可靠。全面推行行政执法三项制度，完善政府法律顾问机制，办理行政复议案件2945件。创建珠三角法治政府示范区，荣获中国法治政府奖。

加强服务型政府建设。坚决落实中央八项规定及其实施细则精神，大力整治庸政懒政怠政和不作为、乱作为。开展政府机构改革。推进“数字政府”建设，完善“一窗式”集成服务、清单管理、跨城通办、一网通办等措施，推行审批服务“马上办、网上办、就近办、一次办”，打造“花城事好办”政务服务品牌。构建热线标准化体系，解决市民诉求129.6万件。推进政务服务、政务公开国家级试点，推动重大项目批准和实施、公共资源配置、社会公益事业建设等重点领域政府信息公开，完善政府会议网络公开机制，政务更加阳光透明。

与此同时，国防动员、双拥共建、海防和打私、打假、妇女儿童、信访、民族宗教、残疾人、侨务、对台、贸促、口岸、气象、地震、民防、参事、文史、档案、保密、供销、修志、仲裁、红十字会等工作取得新成效。

各位代表，过去一年的成绩来之不易。这些成绩的取得，根本在于以习近平同志为核心的党中央坚强领导，在于习近平新时代中国特色社会主义思想的科学指引。这也是认真贯彻落实省委、省政府和市委决策部署的结果，是人大、政协监督支持的结果，是全市人民团结奋斗的结果。我代表广州市人民政府，向全市人民、驻穗部队官兵、各民主党派、人民团体以及社会各界人士表示崇高的敬意！向关心支持广州改革发展的港澳台同胞、海外侨胞、国际友人表示衷心的感谢！

我们也清醒认识到，我市经济社会发展还存在许多不平衡不充分的矛盾和问题。一是经济下行压力较大，产业发展质量不高，传统产业仍占较大比重，新兴产业增速快但体量小，尚未形成有效支撑，投资特别是民间投资不足，民营经济发展面临不少困难，外贸增长乏力。二是企业研发投入比例不高，龙头企业不多，科技成果转化有待加强，高端创新人才、创新团队仍然不足。三是营商环境有待优化，事中事后监管仍需加强，知识产权保护和运用还不能完全适应城市竞争发展要求，现代化国际化水平还不够高。四是城乡治理水平有待提升，违法建设存量大、治理任务重，空气质量持续改善难度较大，黑臭水体治理任务艰巨，城市更新进度仍需加快，城市管理精细化水平还有待进一步提高。五是基本公共服务均等化水平不高，就业、教育、医疗、住房、养老、家政等方面与群众期待还有差距。六是政府职能转变仍需加强，有的工作落实不够到位，有的政府工作人员缺乏服务意识和责任担当，群众办事还有诸多不便，一些领域腐败案件时有发生。对这些问题，我们要采取坚决有力措施加以解决。

二、2019年工作安排

今年是新中国成立70周年，是全面建成小康社会关键之年。做好政府工作，我们必须以习近平新时代中国特色社会主义思想为指导，全面贯彻党的十九大和十九届二中、三中全会以及中央经济工作会议精神，全面贯彻落实习近平总书记对广东重要讲话和对广东工作一系列重要指示精神，落实省委十二届六次全会和市委十一届六次全会要求，统筹推进“五位一体”总体布局，协调推进“四个全面”战略布局，坚持稳中求进工作总基调，坚持新发展理念，坚持推动高质量发展，坚持以供给侧结构性改革为主线，坚持深化市场化改革、扩大高水平开放，扭住粤港澳大湾区建设这个“纲”，统筹推进稳增长、促改革、调结构、惠民生、防风险、保稳定各项工作，进一步稳就业、稳金融、稳外贸、稳外资、稳投资、稳预期，继续打好三大攻坚战，坚持不懈推进全面从严治党，保持经济持续健康发展和社会大局稳定，努力实现老城市新活力，在综合城市功能、城市文化综合实力、现代服务业、现代化国际化营商环境方面出新出彩，推动国家中心城市建设全面上新水平，着力建设国际大都市，在全省实现“四个走在全国前列”、当好“两个重要窗口”中勇当排头兵，以优异成绩庆祝中华人民共和国成立70周年。

综合分析国内外形势，我们看到，广州发展面临的机遇与挑战并存。虽然世界经济延续复苏态势，但受保护主义等多重因素影响，经济下行风险逐步加大。我国发展面临的环境将更加复杂，不确定性也将更大。当前，我市发展正处于新旧动能转换的攻关期，到了滚石上山、爬坡过坎的关键时期，面临许多风险与挑战。但同时我们也要看到，我国发展仍处于并将长期处于重要战略机遇期，国家坚定不移推动高质量发展，加快“一带一路”和粤港澳大湾区建设，加上我市产业体系完备，创新创业活跃，综合优势明显，有能力有条件实现更高质量、更有效率、更加公平、更可持续的发展。

今年经济社会发展主要预期目标是：经济运行稳中有进，地区生产总值增长6－6.5％，固定资产投资增长8％左右，社会消费品零售总额增长7％左右，商品进出口总值增长3％左右。经济高质量发展迈出新步伐，先进制造业增加值占规模以上制造业比重达到68％左右，现代服务业增加值占服务业比重达到68％左右，研发经费支出占地区生产总值比重达到2.8％，地方一般公共预算收入增长5％。人民美好生活质量持续提升，城乡居民收入增长与经济增长基本同步，城市居民消费价格涨幅控制在3％左右，城镇登记失业率控制在3.5％以内。生态环境建设取得新进展，万元地区生产总值能耗下降3％，减排约束性指标完成年度任务。金融财政风险有效防控。为实现上述目标，我们将着力在以下方面狠抓落实：

（一）全面深化改革开放。弘扬敢闯敢试、敢为人先的改革精神，创造性地贯彻中央决策部署，发挥市场在资源配置中的决定性作用，更好发挥政府作用，努力实现最优政策组合和最大整体效果，推动形成全面开放新格局。

全力推进粤港澳大湾区建设。充分发挥粤港澳大湾区核心引擎作用，建立健全工作架构，完善穗港、穗澳对接合作机制，探索建立区域政务服务机制，携手港澳建设国际一流湾区和世界级城市群。建设广深港澳科技创新走廊，加快推进“创新四核十三节点”建设，抓紧建设香港科技大学（广州）校区，打造粤港澳大湾区知识城知识创造示范区、科学城制度创新先行区、生物岛生命科学合作区、黄埔港现代服务创新区，与中国教育科学研究院共建粤港澳大湾区教育研究中心，建设大湾区青年创新创业基地，打造粤港澳科技成果转化基地。推进深茂铁路、南沙港铁路、广中珠澳高铁等项目，推动广深港高铁引入中心城区，加快建设深中通道、南中特大桥、莲花山过江通道等重大项目，推动广州地铁线网向佛山等周边城市延伸。加快建设粤港产业深度合作园、粤澳合作葡语系国家产业园等重大合作平台，积极打造穗港澳国际健康产业城、南站商务区、临空经济示范区、庆盛枢纽、琶洲数字经济创新试验区等特色发展平台。共建湾区科技金融服务中心、文化中心、国际贸易中心、大数据中心，推进设立广州创新型期货交易所、粤港澳大湾区商业银行，建设国际金融岛。深化从化无规定马属动物疫病区与香港合作，支持香港赛马会建设国际马匹检验检疫监测中心和马术运动综合体。全面加强教育、医疗、养老等合作，共建粤港澳大湾区优质生活圈。

着力打造现代化国际化营商环境。围绕打造全球企业投资首选地和最佳发展地，持续深化营商环境改革3项国家级试点，推动形成3个重点改革区域示范，系统开展投资便利化、贸易便利化、市场监管体制等6项改革，开展不动产登记、缴纳税费、获得电力等11个改革专项行动。支持广州开发区打造国家级营商环境改革创新试验区。继续推进简政放权，再精简和下放一批行政许可备案事项，完善政府部门权责清单。大幅放宽市场准入，降低港澳台会计、教育、文化、医疗、养老等行业准入门槛。建立以“信用＋监管”为核心的新型市场监管体系，形成联合奖惩长效机制。强化知识产权保护，打造全国性知识产权交易中心，严厉打击侵权假冒违法行为。

深入推进重要领域和关键环节改革。全面完成政府机构改革任务。深化投融资体制创新，用好政府债券，完善政府和社会资本合作政策体系，有序推进一批合作项目。推动价格领域改革。继续抓好国资国企改革，发展混合所有制经济，推进资源重组整合，实施职业经理人试点，建立完善差异化薪酬分配和长效激励约束机制，做强做优做大国有资本。深化财政事权与支出责任划分、全过程预算绩效管理和市级预算等改革。强化金融服务实体经济功能，常态化办好重大项目融资对接会，加大力度推动企业上市挂牌，大力发展绿色金融、普惠金融、科技金融，强化金融风险防控。

推动外经贸转型发展。深化服务贸易创新发展，落实银行业、信息服务、法律服务等国家服务贸易试点开放措施，加快服务外包发展。加快发展外贸新业态，深化市场采购贸易方式试点、跨境电商综合试验区建设，支持单机、单船和设备融资租赁，推动平行汽车进口全产业链发展，发展毛坯钻石保税交易、保税展示等新业态。积极应对贸易摩擦，帮助企业做好应对措施。深化国际贸易“单一窗口”、智能通关、数字口岸建设，提升跨境贸易便利化水平。加大招商引资力度，举办国际投资年会等招商推介活动，开展产业链招商，引进世界500强、中国500强企业和占据产业链核心的“单打冠军”“隐形冠军”等高端优质企业。

建设国际交往中心。参与共建“一带一路”，建立与沿线国家和地区的多层次多领域合作机制，加强与沿线国家和地区的产能合作，扩大铁路国际货运班列。落实高水平建设国际交往中心三年行动计划，打造具有影响力的国际会议品牌，办好世界港口大会、中国邮轮产业发展大会、海丝博览会等国际会议。加快推动中日生物医药产业合作园区项目建设。发挥侨务优势，深化交流合作。扩大友城交往，推动设立广州、法兰克福、里昂三城经济联盟，开展国际友城工作40周年系列庆祝活动。

（二）全力推动经济高质量发展。坚持质量第一、效益优先，更加重视发展实体经济，深化供给侧结构性改革，在“巩固、增强、提升、畅通”上下功夫，加快构建现代化产业体系。

着力建设先进制造业强市。出台系列产业扶持政策，用好产业引导基金，扶持先进制造业和战略性新兴产业发展。加大力度推进10大价值创新园区建设，培育若干个千亿级产业新集群，争创制造业高质量发展国家级示范区。推动低效园区和村级工业园提质增效，提高单位面积产出效益。大力培育优质企业，加强对接服务，支持一批重大产业项目加快建设，尽早投产达产。采取技术改造、兼并重组等方式，支持汽车、电子、电力、石化等传统优势产业转型升级。释放数字经济潜能，建设4K超高清视频示范社区，推进5G技术研发与商用，加快互联网协议第6版升级改造。加快发展工业互联网，建设省级示范基地、工业互联网标识解析国家顶级节点，加快建设国家级智能网联汽车与智慧交通应用示范区，推动工业企业上云上平台。提前布局量子通信、航空维修、轨道交通、天然气水合物开采及衍生技术等前沿产业。发挥好船舶和电子等军工领域传统优势，支持中电科华南电子产业园和电子五所总部加快建设，促进军民融合发展。大力发展海洋经济。

着力建设现代服务业强市。做优做强高端现代服务业，支持发展法律、会计、咨询、广告、精算、人力资源等专业服务业，建设国际工业设计中心，发展供应链管理、定制化服务、总集成总承包、信息增值服务等服务型制造新业态。加快国际金融城、琶洲互联网创新集聚区等项目建设。推动专业批发市场转型升级，优化物流体系规划建设，开展物流园区整治提升行动。打造国际会展之都，加快推进广交会第四期展馆、琶洲会展塔综合体、物流轮候区、客运口岸码头建设，积极引进国际品牌展会活动落户。发展邮轮经济，加快建设国际邮轮母港。

着力释放内需潜力。发挥投资的关键性作用，大力推进重大项目“攻城拔寨”行动，加快推进592个市重点项目建设，完成投资3087亿元、增长17.6％。努力打造国际消费城市，推动高品质步行街和商圈建设，加大保育、养老、医疗、文化、教育、旅游等服务供给，加快新零售业态布局，扶持“老字号”发展，办好迎春花市、广府庙会及国际灯光节、美食节、购物节等特色品牌活动。深入实施质量强市战略，开展质量提升行动，建好用好高端检验检测公共服务平台，提升产品和服务质量。

着力发展民营经济。落实省“民营经济10条”，优化市“民营经济20条”，搭建跨部门涉企政策“一站式”发布平台，实行政策兑现“一门式”办理。坚决破除各种隐性壁垒，加快放开民间资本进入公用事业和战略性新兴产业等领域，支持民间资本参与国有企业改制重组。清理和精简涉企收费，持续减轻企业税费负担。用好中小企业发展基金，完善中小微企业融资再担保体系，搭建花城创新投资平台，强化金融对民营经济的支持力度。弘扬企业家精神，构建“亲”“清”新型政商关系，建立健全领导干部联系民营企业制度，定期办好市长与民营企业家恳谈会，满腔热情积极主动做好服务，提振民营企业投资信心。

（三）加快建设科技创新强市。坚持创新是第一动力，突出企业创新主体地位，扭住基础研究和关键核心技术攻关两大突破点，完善科技体制机制，共建粤港澳大湾区国际科技创新中心。

完善技术创新体系。加强基础与应用基础研究，建立健全基础科学研究支持体系，组织重大科技专项，参与国家和省重大科技项目研发。壮大科技创新企业，推动一批规模以上企业升级为高新技术企业，支持更多高新技术企业壮大为规模以上企业和行业标杆企业。完善金融支持体系，用好科技成果产业化引导基金，扩大科技信贷风险补偿资金池规模，支持科技企业在境内外上市。完善科技成果转移转化体系，深入开展高校、科研机构科技成果转移转化，加快建设华南（广州）技术转移中心。深化科技交流合作，办好创交会、小蛮腰科技大会、官洲论坛等创新创业活动，加强双创文化建设，打造全球科技活动交流中心、展示中心和交易中心。

建设科技创新平台。加强综合性国家科学中心建设，加快建设再生医学与健康省实验室、南方海洋科学与工程省实验室、新一代通信与网络创新研究院等省级重大创新平台，抓紧建设人类细胞谱系大科学研究设施、冷泉生态系统大科学装置、新型地球物理综合科学考察船等国家级项目，推动国家级大科学装置、大科学研究中心布局落户。促进科技与产业融合，加快建设国家先进高分子材料产业创新中心，组建3至5个国家级产业创新中心、企业技术中心、国地联合工程实验室和省工程实验室。

打造国际人才新高地。出台更具竞争力的人才政策，办好海交会等引智平台，更好发挥“人才绿卡”聚才效应，高标准建设国家级人力资源服务产业园，建设南沙国际化人才特区，加快推进设立海外人才工作站，着力引进一批站在世界科技前沿、处在创新高峰期的领军人才和创新团队。完善人才培养、评价和激励机制，探索实施顶尖人才“全权负责制”，落实科研人员发明成果转化创业、科技成果收益分配、股权期权激励等政策，推动科研机构与企业双向交流，培养一批为企业提供智力支持与服务的科研人才团队。

（四）系统提升综合城市功能。坚持统筹兼顾、优势互补，优化经济发展空间布局，加快枢纽型网络城市建设，以绣花功夫推进城市精细化管理，发挥中心城市集聚辐射带动作用。

建设现代化交通基础设施。推动广州港深水航道拓宽工程二期、南沙港区四期、国际邮轮码头等航运枢纽工程建设。启动白云机场三期扩建工程建设，规划建设白云机场枢纽一体化集疏运体系。推进3个国铁、7个城际轨道、13个地铁、6个综合交通枢纽、9个高速公路、6个城市快速路项目和一批市政工程建设。开工建设深茂铁路、广湛高铁、空港大道三期、如意坊放射线二期等项目，完成广清城际清远至广州北段、地铁21号线（员村—镇龙西段）、南大干线广汽段等项目。完善智慧交通体系，加强道路交通微循环，打通断头路。完善慢行系统。加快综合管廊、停车场、充电桩等市政配套设施规划和建设。

提升城市功能品质。推动“多规合一”深化应用，继续优化一批重点功能区规划。加大精准供地力度，推进批而未供和闲置土地专项整治。加快城市更新改造，推动151个老旧小区微改造、10个旧村改造、50个旧厂改造项目。开展白云山、越秀山“还绿于民”“还景于民”专项整治行动，开工建设广州花园。恩宁路二期（骑楼部分）改造、海珠广场提升优化、芳村大道南快捷化改造3项工程9月底前完工。坚决打好治理违建攻坚战，拆除违法建设4000万平方米以上。推进创建市级容貌示范社区、市容环境卫生责任区示范街（镇）。深入推进“厕所革命”，新改建环卫、旅游、乡村及其他公厕630座。新增管道燃气居民覆盖用户35万户。加强信息基础设施建设，推动智慧灯杆试点，建设国家大数据综合试验区。推进海绵城市建设，加快珠江流域堤岸达标加固工程，提高城市防洪排涝水平。

推进跨区域合作共建。落实省委关于构建“一核一带一区”新格局的部署，强化国家中心城市担当，辐射带动珠江—西江经济带和粤北生态发展区创新绿色发展。推进更高层次的广佛同城化，强化广佛地区粤港澳大湾区增长极建设。共建广清产业协同发展基地，建设广清特别合作区。深入推进穗莞、穗中合作，促进珠江东西两岸融合发展。与齐齐哈尔共建产业园。全力助推毕节、黔南脱贫攻坚，做好疏附、波密、甘孜、巫山和梅州、清远对口帮扶、对口支援，坚决打赢脱贫攻坚战。

（五）扎实推进乡村振兴战略。坚持农业农村优先发展，补齐乡村发展短板，保持乡村本底本色，促进城乡融合，打造全省乡村振兴示范区，努力在全省乡村振兴中当好示范和表率。

发展都市型现代农业。推进高标准农田建设，大力推进现代农业产业园建设，抓好“菜篮子”工程，发展乡村旅游，培育民宿等新业态，促进一二三产业融合发展。建立10个农业科技示范基地和若干农业科技示范户。推进北部山区30个特色小镇建设。大力扶持种业发展，推动国际种业中心建设。推进助农服务平台建设。实施粤菜师傅工程，继续实施“百团千人科技下乡”“千企帮千村”工程，鼓励引导社会人才和各类企业投身乡村振兴。

推进美丽宜居乡村建设。高质量建设岭南特色美丽乡村示范群，大力整治农村人居环境。递减化肥农药使用，推进农用地土壤污染防治，综合治理养殖污染，加强农业生态环境保护。农村水利工程开工35宗，完工25宗。继续推进实施“四好农村路”建设，完善城乡客运网络。继续抓好美丽乡村、中心镇、名镇名村和南粤古驿道建设。

深化农村综合改革。做好农村土地确权成果应用转化，完善农村承包地“三权分置”和农村土地承包经营权流转纠纷调处机制。稳步推进农村集体产权制度改革，完善产权流转服务平台，规范农村集体资产交易行为。完善农村医疗、教育、养老、就业创业服务体系，提升城乡基本公共服务均等化水平，增强农民的获得感、幸福感。

（六）强力推进生态文明建设。牢固树立绿水青山就是金山银山理念，坚决打赢污染防治攻坚战，让广州天更蓝、地更绿、水更清、环境更优美。

坚决打赢黑臭水体剿灭战。持续开展强化水污染防治、剿灭黑臭水体等专项行动，力争13个国考、省考断面达到考核要求。以流域为体系、网格为单元，持续深入开展源头减污工作。全面整顿“散乱污”场所，分类整治养殖场等面源污染。实施河更美大行动，推动落实省“万里碧道”工程。持续推进城中村截污纳管，开工新建扩建17座污水处理厂，力争建成5座，新增污水处理能力48万吨/日，新增1500公里以上污水管网。深入开展320个行政村（社区）农村生活污水治理，持续提升147条河涌水质，推动排水单元达标创建，提升污水收集处理效果。

巩固扩大大气污染治理攻坚战成果。实施煤炭消费减量替代三年行动计划，推进燃煤发电机组节能改造和燃煤锅炉淘汰或清洁能源改造，组织重点企业错峰减排。推进天然气利用四期工程和液化天然气应急调峰气源站项目建设。强化扬尘污染控制，持续开展柴油货车、船舶超标排放专项治理，推动船舶强制使用岸电。在划定区域禁止使用高排放非道路移动机械。加强挥发性有机物污染控制，加大机动车维修、家具制造等行业全过程污染整治力度。完善空气重污染应急预案。全市PM2.5平均浓度控制在35微克/立方米以下。

加快绿色低碳循环发展。完善公交电动化及其配套设施。加强污染地块再开发利用和土壤污染防治。推动垃圾强制分类扩面，创建600个生活垃圾分类样板居住小区（社区），全面建成投产5座资源热力电厂，建成兴丰应急填埋场二期和李坑、福山生物质综合处理厂。推动第三至第七资源热力电厂二期和战略储备填埋场建设。推进废旧资源回收处理体系建设。加快危险废弃物和医疗废弃物处置设施建设。开展建筑垃圾治理试点城市建设。大力发展装配式建筑。

持续加强生态保护和建设。实施主要道路和桥梁绿化提升改造工程，完善城市公园、森林公园景观和服务设施。种植5万株开花乔木，新增赏花点10个。举办国际花卉艺术展、园林博览会。推进海珠湿地品质提升工程，推动森林经营和碳汇造林样板基地建设，新建生态景观林带40公里、森林公园1个、湿地公园1个，新增绿道55公里，创建森林小镇4个，绿化美化80条乡村。持续优化北部绿色生态屏障体系。

（七）加强民生保障和社会治理创新。以增进民生福祉作为发展的根本目的，用心用情多办民生实事、多解民生之忧，努力把广州建设成为全国最安全稳定、最公平公正、法治环境最好的地区之一。

提高社会保障水平。深化“双到双零”就业服务，加强职业技能培训，新增就业人数20万人，重点解决大学毕业生、农民工、退伍军人就业。建立健全基本公共服务标准体系。落实社会保险降费减负政策，稳步提高参保人待遇水平。提高城乡居民医保住院支付比例和大病保障额度，探索全面推进长期护理险。进一步提高城乡居民医保和城乡居民养老保险基础养老金政府补助标准。实施跨省异地就医住院费用即时结算。深化养老服务改革创新，大力推进养老产业发展。优化提升长者助餐配餐服务，把重度残疾人和医疗、护理、心理等服务纳入配餐体系，实现全市镇（街）、村（居）全覆盖。推进医养结合，建设一批养老设施，推动“家政＋养老”融合发展。稳步提高社会救助标准，推行困境儿童分类救助帮扶，试点医疗救助全城通办。扎实做好困难群众法律援助工作。深化创建慈善城市行动。坚持房子是用来住的、不是用来炒的定位，落实城市主体责任，健全住房市场体系和住房保障体系，构建房地产市场平稳健康发展长效机制。加快保障性安居工程建设，新开工棚户区改造1.38万套，基本建成保障房1.2万套。加大公租房保障力度，建立健全公共租赁住房、共有产权住房制度，做好户籍中等偏下住房困难家庭兜底性保障，逐步改善新市民住房条件。培育发展住房租赁市场，推进住房租赁立法，强化住房租赁企业监管，完善租购并举住房制度。

发展均衡而有质量的教育。扩大优质基础教育学位供给，实施发展学前教育第三期行动计划，增加公办幼儿园学位3.8万个，加快实施校园功能微改造，新认定市示范性普通高中10所。深化教育体制机制改革。支持和规范民办教育发展。加快科教城建设，支持技师学院纳入高职院校序列。支持广州大学、广州医科大学高水平大学建设，华南理工大学广州国际校区启动招生，筹建广州交通大学，加快中国科学院大学广州学院建设。坚持立德树人，开展中小学生智慧阅读活动，加强中小学生心理健康教育，大力发展校园足球。深入推进智慧教育创新发展。提高教育国际化水平。深化校外培训机构常态化治理。完善老年教育体系。

加快建设文化强市。培育和践行社会主义核心价值观，构建中华优秀传统文化体系，传承弘扬岭南文化。深化文明城市创建，发展志愿服务事业。加强历史文化遗产保护，编制文物保护规划，推进海上丝绸之路史迹保护和联合申遗工作。制订历史建筑利用修缮监管办法，活化利用北京路、东山、沙面、黄埔古港等重点历史文化片区。推进“图书馆之城”“博物馆之城”建设，加快广州文化馆、美术馆、粤剧院、博物馆新馆、科学馆、音乐博物馆等重点文化设施项目建设。办好中国音乐金钟奖、文化产业交易会、海丝博览会、国际纪录片节、亚洲美食节等一批重大国际性文化活动。开展市民文化节系列活动，加强文艺精品创作，推进文化惠民工程，振兴粤剧艺术。实施文化产业“互联网＋”，壮大数字内容、动漫游戏等新型文化产业，打造百亿级骨干文化企业。推进商旅文融合发展，创建国家文化与金融合作示范区，建设国家全域旅游示范区、北京路国家级文化产业园区，推动广州传统中轴线高标准高质量提升。加快推进体育名城建设，广泛开展全民健身活动，办好国际国内重大体育赛事，创新发展体育产业，做强各区体育品牌。

高质量推进健康广州建设。继续深化公立医院改革，建立现代医院管理制度，加快分级诊疗制度建设。推进高水平医院和临床重点专科建设。提升全科医生服务能力，提高家庭医生签约服务规范化水平。动态调整优化医疗服务项目价格，提高基层诊疗医保支付比例。推进智慧医疗体系建设。完善疾病防控应急体系，做好重大传染病等突发公共卫生事件防控，严防重大疫情发生。市第八人民医院新址二期投入使用，加快市呼吸中心、胸科医院等医疗设施建设。加强中医药强市建设。广泛开展爱国卫生运动，迎接国家卫生城市复审。

扎实做好其他各项社会事业。加强城市民族工作，积极引导宗教与社会主义社会相适应。加强能源和粮食储备建设。扎实做好国防动员、拥军优抚安置、民防、海防、打私、打假、应急、气象、地震、参事、文史、档案、保密、修志、区划等工作。

营造共建共治共享社会治理格局。借鉴北京“街乡吹哨、部门报到”改革经验，以党建引领基层治理创新。推进社区共建共治共享服务中心和社区基金试点，深化社会组织党建工作和“社工＋”战略，推进社区、社会组织、社工“三社联动”建设。深化“四标四实”应用，完善社区网格化管理事项处理机制。积极探索“电梯托管管家”和电梯安全事务社区治理模式。强化出租屋安全隐患整治，持续推进来穗人员融合行动，完善积分制公共服务提供机制。加强预防和化解社会矛盾机制建设，加大信访积案化解处理力度。创建全国公共法律服务最便捷城市。夯实党政领导安全生产责任，健全风险隐患排查整治闭环机制，建设应急救援和风险管控综合体系，提高自然灾害防治能力。全面实施食品安全战略。建设平安广州，铁腕推进扫黑除恶专项斗争，严密防范暴力恐怖活动，严厉打击各类刑事犯罪，深入开展社会治安重点地区和突出问题排查整治，努力提高社会治理智能化、科学化、精准化水平。

（八）全面加强政府自身建设。我们要强化“四个意识”，坚定“四个自信”，坚决做到“两个维护”。深入推进政府职能转变，体现人民意愿，维护人民利益，全面提升履职水平，为人民提供优质高效服务。

规范行政权力运行。落实全面依法治国要求，严格在宪法和法律的范围内行使权力，加快法治广州建设步伐。深入推进科学民主依法立法，提高重大行政决策质量。深入推进行政执法三项制度，发挥行政执法监督系统作用，促进严格规范公正文明执法。推进行政复议体制改革，发挥行政复议解决行政争议的主渠道作用。完善行政调解、行政裁决、仲裁制度，依法化解社会矛盾，促进社会和谐。

全面加强党风廉政建设。深入落实中央八项规定及其实施细则精神，贯彻全面从严治党要求，把党风廉政建设和反腐败工作不断引向深入。加强审计监督，抓好审计整改工作。严肃查处侵害群众利益的不正之风和腐败问题，始终保持廉洁本色，营造风清气正干事创业的良好政治生态。弘扬求真务实的作风，加强作风建设。健全激励机制和容错纠错机制，严格工作责任制，激励广大干部在新时代改革开放中有新担当新作为，严厉整肃庸政懒政怠政行为。

提高政务服务水平。全面推进模范机关建设。深化政务服务创新，优化办事流程，坚决治理政务失信，不断提升人民群众对政务服务的满意度。全面推动“数字政府”建设，加强信息互联互通。深化政务服务便民化工作，强化政务服务规范化标准化建设，优化政府服务热线大数据分析和应用，规范网上中介服务超市运行，重点领域和常办事项基本实现“一网通办、只进一扇门、最多跑一次”。

主动接受监督和及时回应社会关切。依法接受市人大及其常委会的监督，自觉接受市政协的民主监督，广泛听取人大代表、政协委员、民主党派、工商联、无党派人士和各人民团体的意见，认真办理人大代表议案建议和政协提案。建立健全常态化的改革督察机制，加强改革实施效果评估，以实实在在的改革成效惠及广大市民群众。加大政务公开力度，加强对涉及民生、产业发展、营商环境等重大政策措施的宣传和解读。每一位政府工作人员特别是领导干部，既要雷厉风行，又要久久为功，以钉钉子的精神做实做细做好各项工作，努力做人民满意的公务员。

各位代表，幸福是奋斗出来的，奋斗才能赢得未来。让我们高举习近平新时代中国特色社会主义思想伟大旗帜，在省委、省政府和市委的坚强领导下，推动思想再解放、改革再深入、工作再抓实，推进国家中心城市建设全面上新水平，着力建设国际大都市，以优异成绩庆祝中华人民共和国成立70周年，为实现中华民族伟大复兴的中国梦作出新的更大贡献！
